# Experimental infection of a US spike-insertion deletion porcine epidemic diarrhea virus in conventional nursing piglets and cross-protection to the original US PEDV infection

**DOI:** 10.1186/s13567-015-0278-9

**Published:** 2015-11-20

**Authors:** Chun-Ming Lin, Thavamathi Annamalai, Xinsheng Liu, Xiang Gao, Zhongyan Lu, Mohamed El-Tholoth, Hui Hu, Linda J. Saif, Qiuhong Wang

**Affiliations:** Food Animal Health Research Program, Ohio Agricultural Research and Development Center, College of Food, Agricultural and Environmental Sciences, Department of Veterinary Preventive Medicine, The Ohio State University, Wooster, OH USA; State Key Laboratory of Veterinary Etiological Biology, OIE/National Foot-and-Mouth Disease Reference Laboratory of China, Lanzhou Veterinary Research Institute, Chinese Academy of Agricultural Sciences, Lanzhou, China; Department of Virology, Faculty of Veterinary Medicine, Mansoura University, Mansoura, Egypt

## Abstract

Although the original US porcine epidemic diarrhea virus (PEDV) was confirmed as highly virulent by multiple studies, the virulence of spike-insertion deletion (S-INDEL) PEDV strains is undefined. In this study, 3–4 day-old conventional suckling piglets were inoculated with S-INDEL PEDV Iowa106 (4 pig litters) to study its virulence. Two litters of age-matched piglets were inoculated with either the original US PEDV PC21A or mock as positive and negative controls, respectively. Subsequently, all pigs were challenged with the original US PEDV PC21A on 21–29 days post-inoculation (dpi) to assess cross-protection. All S-INDEL Iowa106- and the original US PC21A-inoculated piglets developed diarrhea. However, the severity of clinical signs, mortality (0–75%) and fecal PEDV RNA shedding titers varied among the four S-INDEL Iowa106-inoculated litters. Compared with the original PC21A, piglets euthanized/died acutely from S-INDEL Iowa106 infection had relatively milder villous atrophy, lower antigen scores and more limited intestinal infection. Two of four S-INDEL Iowa106-infected sows and the original PC21A-infected sow showed anorexia and watery diarrhea for 1–4 days. After the original PC21A challenge, a subset (13/16) of S-INDEL Iowa106-inoculated piglets developed diarrhea, whereas all (5/5) and no (0/4) pigs in the mock and original PC21A-inoculated pigs had diarrhea, respectively. Our results suggest that the virulence of S-INDEL PEDV Iowa106 was less than the original US PEDV PC21A in suckling pigs, with 100% morbidity and 18% (6/33) overall (0–75%) mortality in suckling pigs depending on factors such as the sow’s health and lactation and the piglets’ birth weight. Prior infection by S-INDEL Iowa106 provided partial cross-protection to piglets against the original PC21A challenge at 21–29 dpi.

## Introduction

Porcine epidemic diarrhea (PED) is a highly contagious swine enteric disease resembling transmissible gastroenteritis (TGE). It was first recognized among English feeder and fattening pigs in 1971 [1]. Experimental inoculation with the Belgian isolate, the PED virus (PEDV) prototype CV777 strain, revealed that PEDV is enteropathogenic for both nursing and fattening pigs [2]. Subsequently, the etiological agent of PED was identified as PEDV, belonging to the *Alphacoronavirus* genus within the *Coronaviridae* family. Before the end of 2010, endemic PED had been reported in many European and Asian countries, but with low impact. The subsequent PED pandemic outbreaks started in China [3] and spread to other Asian counties, inducing high piglet mortality [4]. In April 2013, PED outbreaks occurred suddenly in US swine [5]. Piglets younger than 7 days old developed vomiting and diarrhea that led to dehydration, rapid weight loss, and death in 2–4 days, with mortality reaching 100% in suckling piglets [6]. PEDV infection also impaired the performance of surviving pigs [7], resulting in significant economic losses to the US pork industry.

Sequence analysis of complete PEDV genomes revealed that the original US PEDV strains were closest to one recent Chinese strain AH2012 [8]. The high virulence of the original US PEDV strains was experimentally confirmed in gnotobiotic (Gn) piglets [9], cesarean-derived and colostrum-deprived (CDCD) piglets [10, 11] and conventional piglets [12, 13]. Also, compared with neonatal piglets, conventional 3–4 weeks old pigs were less susceptible to the original US PEDV infection [12–14]. Concurrently, several US variant PEDV strains, characterized by insertions and deletions (INDELs) in the spike (S) gene and designated as S-INDEL PEDV, were found to be circulating in US swine farms [8, 15]. When compared with three other original US PEDV strains (USA/IN/2013/19338P7, USA/NC/2013/35140P7, and USA/NC/2013/49469P7), 5-day-old non-suckling piglets inoculated with an S-INDEL PEDV strain (USA/IL/2014/20697) developed no clinical signs and mild histopathologic lesions [16]. The virulence of the S-INDEL PEDV in pigs in the field varied. In one report, S-INDEL PEDV OH851 strain infected pigs showed minimal to no clinical signs in pigs in the field [15]. However, recent reports described that European S-INDEL PEDV strains caused high mortality in suckling piglets in southern Germany [17] and southern Portugal [18]. Sequence analysis of partial S1 gene revealed that the Portugal PEDV strain shared 99 and 100% nucleotide identities with US S-INDEL PEDV OH851 strain and the German strains (GER/L00719/2014 and GER/L00721/2014), respectively [18]. Factors contributing to the contradictory clinical signs have not yet been clarified [19].

We recently reported that convalescent antisera obtained from S-INDEL-infected pigs cross-reacted with the original US PEDV PC22A strain in two-way cell culture immunofluorescence and viral neutralization assays [20]. Others reported that sows recovered from natural infection by an S-INDEL PEDV, 7 months prior to farrowing and orally boosted with an original US PEDV strain around 109 day of gestation provided lactogenic immunity and partial protection to their piglets from a subsequent challenge by an original US PEDV [21]. These in vivo and in vitro antigenicity and cross-protection studies suggest that S-INDEL PEDV strains may serve as vaccine candidates to protect pigs from highly virulent original US PEDV strains [20, 21], if the S-INDEL PEDV strains are confirmed as naturally attenuated strains. Our aims were to evaluate the pathogenicity of S-INDEL PEDV Iowa106 strain in conventional suckling piglets and to examine whether infection of the piglets with this S-INDEL PEDV strain induces cross protection against diarrhea caused by a subsequent (3–4 weeks later) challenge with the original US PEDV PC21A strain.

## Materials and methods

### PEDV inoculum

Pig intestinal contents containing the S-INDEL PEDV Iowa106 (GenBank accession no. KJ645695) were collected from a pig during a mild diarrhea outbreak [8]. The original sample tested negative for group A, B and C rotaviruses at the Veterinary Diagnostic Laboratory, University of Minnesota, and TGEV/porcine respiratory coronavirus and porcine deltacoronavirus in our laboratory as described previously [9, 22]. Because the volume of the original field PEDV sample containing PEDV Iowa106 strain was very limited, the virus was directly passed once in one conventional pig litter (litter A) to generate a virus pool. The intestinal contents collected from one piglet at 3 dpi were stored in aliquots at −80 °C and used to prepare inocula for the following 3 litters (litters B, C and D). The intestinal contents were suspended in cell culture grade phosphate buffered saline (PBS; pH 7.4; Sigma-Aldrich, St. Louis, MO, USA) followed by vortexing and centrifugation at 2095 × *g* for 30 min. The supernatant was collected and diluted further or filtered through 0.22 μm-pore size filters before using as inocula. The original US PEDV PC21A was collected from the intestinal contents of a 1-day-old diarrheic field pig and passaged twice in Gn piglets [9]. Based on prior experience [9, 10, 23], the viral infectious titers in PFU were about 6 log_10_-lower than the RNA titers and PEDV infectious titer decreased about 1 log_10_ after one freeze–thaw cycle or ultrafiltration (unpublished data). Therefore, the doses of each inoculum (approximately 3–4 log_10_ PFU/pig) were adjusted to the comparable titers of the 10 log_10_ genomic equivalents (GE) (frozen and thawed once, without filtration) according to the inoculum preparation process (Table 1). The PBS was used as a mock control (litter F). In addition, no cross-contamination between original US PEDV PC21A and S-INDEL PEDV Iowa106 in each inoculum was confirmed by conventional differential RT-PCR with PEDV strain-specific primers, which amplified different sizes for the original US and S-INDEL PEDV strains (Liu and Wang, unpublished data).

**Table 1:** General litter information and the clinical signs of piglets and sows after PEDV inoculation (before challenge)

Litter no.	Litter size; stillborn; lost to injury	Age (day); body weight (kg) at inoculation	Inoculum strain (passage)^D^; dose (log_10_ GE/pig); processing method	Piglet condition									Sow condition		
				Morbidity (%)^A^	Mortality (%)^A^	Highest fecal PEDV shedding titer (log_10_ GE/mL)	Onset of diarrhea (dpi)	Duration of diarrhea (days)^C^			Duration of hypothermia (days)^C^	First week mean body weight gain (kg)^C^	Anorexia	Diarrhea (RS ≧ 2)	Highest fecal PEDV shedding titer (log_10_ GE/pig)
								RS = 3^B^	RS ≧ 2^B^	RS ≧ 1^B^					
A	7; 0; 1	3; NA	Iowa106 (P0); 12; F&T 2×, filtrated	100 (6/6)	0 (0/5)	11.24 ± 1.80^a,b^	1	1.80 ± 0.45^b^	4.20 ± 0.84^c^	7.00 ± 0.71^a,b^	NA	NA	0	0	9.46
B	14; 3; 1	3; 1.67 ± 0.31^b^	Iowa106 (P1); 10; F&T 1×,	100 (10/10)	75 (6/8)	11.08 ± 0.05^b^	2	4.67 ± 0.58^a^	6.33 ± 0.57^a,b^	8.33 ± 1.53^a^	6.75 ± 1.71^a^	−0.28 ± 0.16^c^	4	4	11.08
C	12; 1; 1	4; 1.8 ± 0.10^b^	Iowa106 (P1); 12; F&T 2×	100 (10/10)	0 (0/10)	12.67 ± 0.48^a^	2	4.00 ± 1.11^a^	5.78 ± 0.97^b^	6.56 ± 0.73^b^	1.11 ± 0.93^c^	−0.1 ± 0.20^c^	1	2	11.60
D	11; 0; 1	4; 2.2 ± 0.30^a^	Iowa106 (P1); 12; F&T 2×	100 (10/10)	0 (0/10)	12.17 ± 0.47^a^	2–3	1.56 ± 0.73^b^	3.44 ± 1.01^c^	6.00 ± 1.41^b^	0.00 ± 0.00^d^	0.5 ± 0.30^b^	0	0	9.40
E	13; 2; 0	4; 1.60 ± 0.42^b^	PC21A (P2); 10; F&T 1×	100 (11/11)	55 (6/11)	11.80 ± 0.89^a^	1	5.25 ± 0.96^a^	7.20 ± 0.45 ^a^	8.25 ± 1.26^a^	3.22 ± 2.17^b^	0.03 ± 0.31^c^	2	5	9.67
F	10; 2; 1	4; 1.80 ± 0.16^b^	Mock	0 (0/7)	0 (0/7)	ND	ND	0.00 ± 0.00^c^	0.00 ± 0.00^d^	0.00 ± 0.00^c^	0.00 ± 0.00^d^	0.95 ± 0.16^a^	0	0	ND

dpi, days post-inoculation; RS, rectal swab; ND, not detectable; NA, not available; GE, genomic equivalents; F&T, frozen and thaw (once 1x, twice 2x); P1, passage level 1; P2, passage level 2; filtrated, filtrated through 0.22 μm-pore size

^a, b, c, d^ Different letters in each column mean significant different levels among litters (*P* < 0.05).

^A^Piglets injured by their sow or were not moribund when they were euthanized during acute infection phase for histopathology examination were excluded.

^B^RS score: 0 = normal, 1 = pasty, 2 = semi-liquid, 3 = liquid feces.

^C^Piglets died from physical trauma or euthanized for histopathological examination were excluded.

^D^Viruses were passaged in gnotobiotic piglets (the original US PEDV PC21A) or conventional piglets (S-INDEL PEDV Iowa106).

### Animals

Six Large White × Duroc crossbred, pregnant sow (A) or gilts (B, C, D, E, F) at 93 to 100-day of gestation were sourced from a specific pathogen free swine herd of The Ohio State University. The sows/gilts tested seronegative for PEDV by CCIF [20] and ELISA (Annamalai, Saif and Wang, unpublished). All sows/gilts arrived at least 2 weeks before farrowing for adaption to the facility. The sows/gilts farrowed naturally in our biosafety level-2 animal facility. Each pig litter (sow and her piglets) was housed in a separate room. All piglets were evaluated and were healthy on the day of inoculation.

### Experimental design

All animal-related experimental protocols were approved by The Ohio State University Institutional Animal Care and Use Committee. Six conventional sows and their litters (litter A–F) were assigned randomly to three groups: (1) S-INDEL PEDV Iowa106 inoculation (litters A–D); (2) The original US PEDV PC21A inoculation (litter E); and (3) Mock inoculation (litter F). Neonatal suckling piglets were inoculated at 3–4 days of age. Piglets were observed three times daily for the first 7 days post-inoculation (dpi) and twice daily thereafter until the end of the study. Clinical signs, including vomiting, diarrhea, anorexia and depression, were evaluated. Rectal swabs were collected and scored daily for the first 9 dpi and every other day thereafter. Fecal consistency was scored as follows: 0, solid; 1, pasty; 2, semi-liquid; 3, liquid, respectively. The rectal temperatures and body weights were recorded daily for each piglet at 0 (pre-inoculation) to 7 dpi and then weekly thereafter. Sows were considered as anorexic when their feed consumption was reduced ≧50%. If anorexia persisted for more than 2 days, the sows were medically treated with Flunixin meglumine (Banamine^®^, Merck; 10 mL, IM) and Pepto-bismol (P&G Everyday, 60 mL, PO) by the veterinarian to improve their appetite.

One to two piglets in each litter was randomly selected and euthanized for histopathology evaluation at 3 dpi; others, unless they were moribund and fit early removal criteria, were retained to evaluate the duration of clinical signs, mortality and fecal viral shedding.

On the day before the virulent original US PEDV PC21A challenge [day post-challenge (dpc) −1], one pig in each litter was euthanized to observe any histopathological lesions in the pigs that survived the primary acute infection. At 21–29 dpi, all pigs were challenged with the original US PEDV PC21A. The clinical parameters as described earlier were measured/recorded daily. All piglets were euthanized at 7 dpc/28–36 dpi for necropsy examination.

### Gross and histopathological examination

At necropsy, both intestine and other major organs were examined. Duodenum (5 cm distal to the pylorus), jejunum (three samples taken at 40–60 cm intervals), ileum (5 cm anterior to the ileo-caecal valve), cecum, the middle segment of colon and mesenteric lymph nodes were collected. After 48 h fixation in 10% neutral buffered formalin, tissue sections were trimmed, processed, and embedded in paraffin. Four micron sections were cut and routinely stained with hematoxylin and eosin. For each jejunum section, at least ten villi and crypts were measured using a computerized image system with villous height and crypt depth (VH:CD) ratios calculated as previous described [9].

### Immunohistochemistry (IHC) staining

The IHC staining was optimized as described previously [10, 24] using a non-biotin polymerized horseradish peroxidase system (BioGenex Laboratories, San Ramon, CA, USA). The IHC signal of PEDV nucleocapsid (N) protein was scored as 0–3 according to the percentage of villous enterocytes within the section showing a positive signal. Score 0 denotes no positive cells; scores 1–3 denote less than 30%, 30 to 60% and more than 60% of villous enterocytes showing a positive signal, respectively.

### Analysis of PEDV RNA fecal shedding titers

Two rectal swabs were suspended in 4 mL Minimum Essential Media (Invitrogen, Carlsbad, CA, USA) as a 10% fecal suspension. The RNA was extracted from 50 μL of clarified (centrifugation at 2095 × *g* for 30 min at 4 °C) fecal suspensions using MagMax™-96 Viral Isolation kit (Ambion, Austin, TX, USA) according to the manufacturer’s instructions. The PEDV fecal shedding titers were determined by TaqMan real-time reverse transcription-PCR (RT-qPCR) with the primers and probe targeting the conserved N protein region of PEDV as described previously [23]. The detection limit was 10 GE per 20 μL of reaction, corresponding to 4.8 log_10_ GE per mL of the original fecal samples.

### Statistical analysis

Comparison of piglets’ rectal temperatures before and after inoculation was conducted by paired T test. The body weight, duration of diarrhea, fecal PEDV RNA shedding titers among litters were compared using one way analysis of variance (ANOVA) followed by Duncan’s multiple range test. The continuous variables between group 1 (S-INDEL PEDV Iowa106 inoculation) and group 2 (the original US PEDV PC21A inoculation) were compared by student’s *t* test. To compare the weekly body weight gain among pig litters, analysis of covariance (ANCOVA) was applied to adjust initial body weights. In addition, correlations between continuous variables were calculated using Pearson correlation coefficients. Statistical analyses were done using SAS (Statistical Analysis System; SAS for windows 9.12; SAS Institute Inc., Cary, NC, USA). A *P* value of less than 0.05 was considered statistically significant.

## Results

### Clinical signs

The general information and the clinical signs for 6 pig litters are summarized in Table 1. After inoculation with S-INDEL PEDV Iowa106, all piglets developed watery diarrhea (RS = 3) within 3 days. Transient vomiting was noted in one and two piglets in litters B and C, respectively. However, the magnitude and duration of diarrhea differed significantly among these litters. Piglets in litters A and D showed watery diarrhea (RS = 3) for only 1–2 days and diarrhea (RS ≧ 2) subsided within 5 days. Piglets in litters B and C had longer duration (6.33 ± 0.57 and 5.78 ± 0.97 days, respectively) of diarrhea. In addition, for all the piglets, the duration of diarrhea correlated negatively with the piglet body weight measured at the day of inoculation (0 dpi) (r = − 0.26, *P* < 0.01). The most hypothermia and mortality were seen only in litter B: piglet body temperature dropped from 39.02 ± 0.36 °C at 1 dpi to 36.50 ± 0.84 °C at 3 dpi (*P* < 0.01); and 75% (6/8) of the piglets died or were moribund and were euthanized. In litter C, a slight decrease of body temperatures (about 1 °C) was observed only on the day of onset of clinical signs (2 dpi) and no piglets died. Piglets in both litters B and C had a decrease in mean body weight gain (−0.28 ± 0.16 and −0.1 ± 0.20 kg) between 0 and 7 dpi. The piglets in litter D gained 0.50 ± 0.30 kg between 0 and 7 dpi, which was significantly higher than those of litters B and C, but still lower than that of the mock-inoculated litter F (0.95 ± 0.16 kg). Sows B and C, but not sows A and D, had anorexia and watery diarrhea, lasting 2 and 4 days, respectively.

In the original US PEDV PC21A-inoculated litter E, all piglets showed watery diarrhea within 1 dpi. Transient vomiting was also observed in two piglets. The body temperature of piglets dropped dramatically from 39.1 ± 0.2 °C at 0 dpi to 37.2 ± 0.9 °C at 1 dpi (*P* < 0.01). Mortality was 55% (6/11). The surviving piglets had diarrhea for 7.20 ± 0.45 days, which was significant longer than that in the S-INDEL-inoculated piglets (Tables 1 and 2). Compared with non-surviving piglets (*n* = 6), the surviving piglets (*n* = 5) had significantly higher body weight (1.84 ± 0.36 vs. 1.36 ± 0.40 kg) at 4-days of age (0 dpi) (*P* < 0.05). However, they did not gain body weight by 7 dpi. In addition, sow E had anorexia for 2 days and transient diarrhea for 5 days. No clinical signs were observed in piglets of the mock group (litter F).

**Table 2:** Comparison between S-INDEL PEDV Iowa106- and the original US PEDV PC21A-infection in conventional suckling piglets

	PEDV strain	
	S-INDEL Iowa106 (4 litters; *n* = 36)	Original US PC21A (l litter; *n* = 11)
Piglet morbidity	100%	100%
Piglet mortality	18% (0–75%)	55% (NA)
Onset of diarrhea (dpi)	2.06 ± 0.63 (1–3)*	1.00 ± 0.00 (1–1)
Duration of diarrhea (RS ≧ 2; days)	4.75 ± 1.52 (2–7)*	7.20 ± 0.45 (7–8)
Highest fecal PEDV RNA shedding titer (log_10_ GE/mL)^a^	11.67 ± 1.07 (9.46–13.40)	11.76 ± 0.91 (10.03–13.13)
VH:CD ratio in jejunum^b^	2.90 ± 1.24* (1.36–5.40)	1.40 ± 0.47 (0.85–1.98)
PEDV antigen score in jejunum^b^	1.40 ± 0.70* (1–3)	2.50 ± 1.00 (1–3)

Data are showed as mean ± standard deviation (full range).

dpi, days post-inoculation; GE, genomic equivalent; VH:CD, the ratio of villous height:crypt depth; NA, not available.

^a^It was detected on the same day of onset of diarrhea.

^b^Piglets died or euthanized between 2 and 6 dpi.

* Significant difference between Iowa106 and PC21A by student *t* test (*P* < 0.05).

### Fecal PEDV RNA shedding profiles

**Figure 1:**
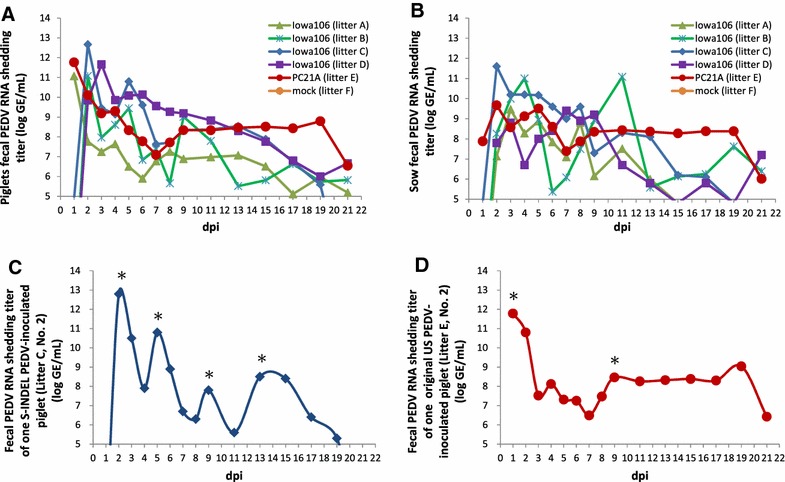
**Fecal PEDV RNA shedding profiles of piglets (A) and their sows (B) after oral inoculation of piglets at 3–4 days of age.**. Data were shown as mean of piglets (**A**) or individual sow (**B**) of each litter. Representative PEDV RNA fecal shedding pattern of one S-INDEL PEDV Iowa106- (**C**) and one original US PEDV PC21A- (**D**) inoculated piglets were shown. A biphasic curve with 3–6 days of intervals between peaks (**C**) or a time-dependent, gradual down-sloping curve (**D**) was observed. A dominant “peak” of fecal PEDV RNA shedding titer was defined when the titer difference between the peak and the lowest values was >1.5 log_10_ (~5 Ct) and was marked with Asterisk. Four and two peaks were counted in (**C**) and (**D**), respectively.

Neither pre- nor mock-inoculated piglets (litter F) shed PEDV RNA in the feces. Among 4 S-INDEL PEDV Iowa106-inoculated litters, the first and also the highest peak titer of PEDV RNA fecal shedding was detected on the day of onset of clinical signs, at 1–3 dpi (Figure 1A). The means of the highest fecal PEDV RNA shedding titers were all above 11 log_10_ GE/mL in all S-INDEL Iowa106-inoculated litters (A–D) (Table 1). Subsequently, the titer gradually decreased but increased again every 3–6 days. For example, piglet No. 2 of litter C had relatively higher (12.8 and 10.8 log_10_ GE/mL) titers at 2 and 5 dpi but lower titers (7.9 and 6.3 log_10_ GE/mL) at 4 and 8 dpi (Figure 1C). Overall, continuous fecal PEDV RNA shedding beyond 21 dpi was detected in a majority (21/26) of surviving piglets.

In litter E, the original US PEDV PC21A group, the highest fecal PEDV RNA shedding titer (11.80 ± 0.89 log GE/mL) was detected on 1 dpi (Figure 1A). Subsequently, the titers gradually dropped to 7.09 ± 0.44 log_10_ GE/mL at 7 dpi and were consistently maintained around 8.0–9.0 log_10_ GE/mL during 9–19 dpi (Figures 1A and D).

In both S-INDEL Iowa106- and the original US PC21A-inoculated litters, all sows were infected with PEDV by direct contact with their piglets (Figure 1B). Generally, higher titers (>8 log_10_ GE/mL) were detected before 14 dpi. Thereafter, the titer dropped in the 4 S-INDEL Iowa106-infected sows (A–D). However, the original US PC21A-infected sow maintained higher fecal virus shedding titers (>8 log_10_ GE/mL) to 19 dpi.

To exclude the possibility of cross-contamination between the original US PEDV PC21A and S-INDEL PEDV Iowa106, the intestinal contents obtained from euthanized piglets and RS obtained from sows of each litter were confirmed as the corresponding PEDV strains by conventional RT-PCR with PEDV strain-specific primers (unpublished data). In addition, the PCR products (the region between nucleotide 20308–21318 based on S-INDEL PEDV Iowa106 strain) were sequenced directly and no nucleotide change was observed before and after one passage in piglets.

### Gross lesions, histopathology and immunohistochemistry staining

During the acute stage of infection (2–4 dpi), 10 S-INDEL PEDV Iowa106- and 4 the original US PEDV PC21A-infected piglets were euthanized for histopathological examination. The lesions incurred by S-INDEL Iowa106 and the original US PC21A could not be distinguished by gross pathological examination. All piglets died/euthanized were emaciated with yellow feces coating the skin and hair. In some piglets, the intestinal lumens were filled with large amounts (approximately 50–70 mL) of yellowish foamy fluid. In other piglets, the walls of the small intestine were transparent and thin and the intestinal lumens were empty. No significant gross lesions were observed in other major organs (lung, kidney, liver and heart).

**Figure 2:**
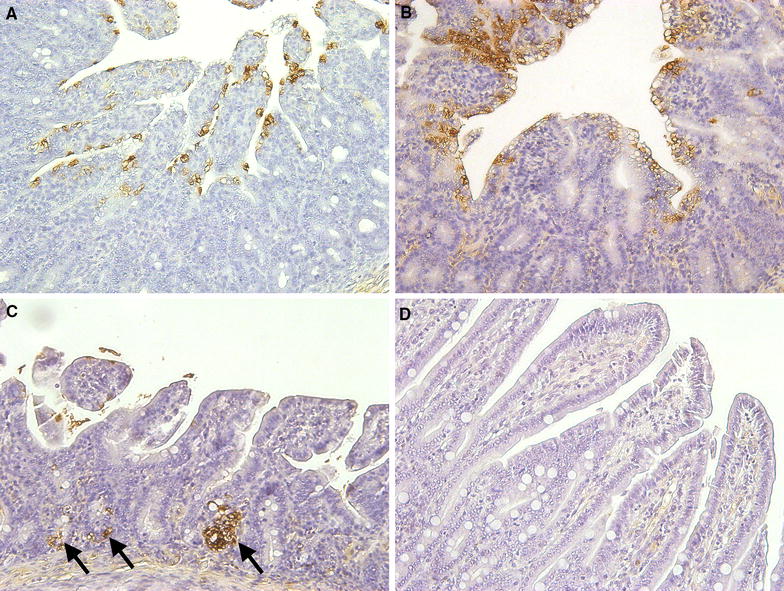
**Antigen distribution pattern of S-INDEL PEDV Iowa106 strain (A), the original US PEDV PC21A strain (B and C), and mock (D) in jejunum.** PEDV nucleocapsid proteins were detected by immunohistochemistry staining (brown) using monoclonal antibody SD6-29 against the N protein of PEDV. Both S-INDEL PEDV Iowa106 (**A**) and the original US PEDV PC21A (**B** and **C**) antigens were mainly detected in villous epithelial cells. Severe villous atrophy was observed in the original US PEDV PC21A-inoculated pigs (**B**). Incidentially, dominant villous atrophy along with the original US PEDV PC21A antigen located in crypts (arrow) were noted in one piglet (litter E, no. 3) (**C**).

**Figure 3:**
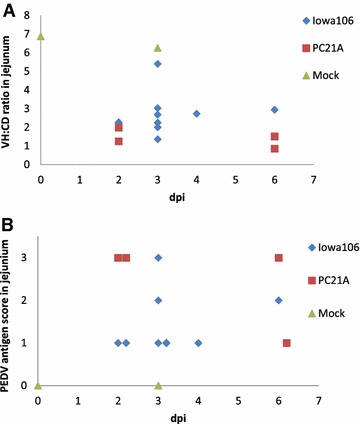
**Histopathology and immunohistochemistry results of piglets that died or were euthanized by 7 days post-inoculation (dpi).** The intensities of villous atrophy and PEDV infection in jejunum were expressed as (**A**) villous high: crypt depth ratios (VH:CD) and (**B**) antigen scores, respectively. Score 0 denotes no positive cells; scores 1–3 denote less than 30%, 30 to 60% and more than 60% of villous enterocytes showing a positive signal, respectively.

Microscopic examination revealed subacute, moderate to severe, extensive, atrophic enteritis in S-INDEL Iowa106-inoculated piglets. Shortening, blunting and fusion of the villi, and occasionally, vacuolization and exfoliation of enterocytes were noted. IHC staining showed brown signal of PEDV N proteins were located in the cytoplasm of villous epithelial cells (Figure 2A). The VH:CD ratios in jejunum ranged between 1.36 ± 0.98 and 5.04 ± 0.58 (Figures 2A and 3A). Except for one piglet (No. 2 of litter B) that had a PEDV antigen score of 3 in jejunum, all piglets (*n* = 10) had scores equal or below 2 in the jejunum (Figures 2A and 3A) and ileum. No PEDV antigen was detected in the crypt cells. Sporadically, PEDV antigens (score = 1) were observed in duodenum in 50% (5/10) of S-INDEL Iowa106-infected piglets. Weak PEDV antigen signal was scattered in the colon of one piglet (No. 7 in litter B).

Microscopic lesions and viral antigen distribution patterns observed in the original US PEDV PC21A-infected piglets (Figures 2B and 3B) were more severe and extensive than those observed in S-INDEL Iowa106-inoculated piglets (Table 2). The VH:CD ratios ranged between 0.85 ± 0.36 to 1.98 ± 0.22 in the four original PC21A-inoculated piglets (Figures 2B, 2C, and 3A). The signal of the original US PEDV antigen was mainly located in epithelial cells covering the villi (Figure 2B) and, occasionally, in some crypt cells (Figure 2C). The virus antigen scores of 3 in jejunum and 2 in ileum were observed in all piglets infected with the original US PEDV (Figure 3B), with the exception of one piglet (No. 3 in litter E). That piglet had a clustered PEDV antigen signal in crypt cells, but less frequent signal in villous epithelial cells (Figure 2C).

No significant gross and microscopic lesions or PEDV IHC antigens were noted in pre-inoculation (0 dpi) or mock-inoculated piglets (litter F). The VH:CD ratios were 6.88 ± 0.12 and 6.26 ± 0.34 in two piglets, respectively (Figure 3).

### S-INDEL PEDV Iowa106 infection induced partial cross-protection in piglets against the original US PEDV PC21A challenge

The clinical signs of pigs after challenge with the original US PEDV PC21A are summarized in Table 3. The PEDV naïve pigs (litter F) were challenged with 10 log_10_ GE/pig of the original US PC21A at 29-days of age. However, no clinical signs were observed by 3 days after challenge, probably due to older pigs being less sensitive to PEDV as reported previously [12–14, 25]. Therefore, these piglets were challenged again with a 2-log_10_ higher dose (12 log_10_ GE/pig) at 32-days of age. Moderate to severe diarrhea, as well as the peaks of fecal PEDV RNA shedding (10.57 ± 0.81 log_10_ GE/mL) occurred at 2 or 3 dpc in all (5/5) pigs. Diarrhea lasted 3.80 ± 0.84 days and completely subsided by 7 dpc in all pigs.

**Table 3:** Clinical signs of pigs after challenge with the original US PEDV strain PC21A

Litter no.	Inoculation piglets at 3–4-days of age	Challenge strain (passage)^C^; dose (log_10_ GE/pig)	Age (day) at challenge (dpi)	Piglet condition								Sow condition		
				Morbidity (%)	Mortality (%)	Highest fecal PEDV RNA shedding titer (log_10_ GE/pig)	Onset of diarrhea (DPC)	Duration of diarrhea (day)			Body weight gain during 0–7 DPC (kg)	Anorexia	Diarrhea (RS ≧ 2)^B^	Highest fecal PEDV RNA shedding titer (log_10_ GE/pig)
								RS = 3^B^	RS ≧ 2^B^	RS ≧ 1^B^				
A	Iowa106	PC21A (P2); 10	24 (21)	0 (0/4)	0 (0/4)	6.21 ± 0.99	–	0.00 ± 0.00	0.00 ± 0.00	0.00 ± 0.00	NA	0	0	5.44
B	Iowa106	PC21A (P2, P4); 10^A^, 12	29, 32 (25, 29)	0 (0/2)^A^	0 (0/2)^A^	7.44 ± 0.10^A^	–	0.00 ± 0.00^A^	0.00 ± 0.00^A^	0.00 ± 0.00^A^	0.85 ± 0.35^A^	0^A^	0^A^	7.65
C	Iowa106	PC21A (P4); 12	27 (23)	86 (6/7)	0 (0/7)	10.8 ± 0.76^a^	1–2	1.00 ± 1.15^a^	2.29 ± 1.60	4.00 ± 1.29	0.93 ± 0.36^c^	0	0	8.00
D	Iowa106	PC21A (P4); 12	27 (23)	100 (7/7)	0 (0/7)	9.61 ± 1.64^a^	1–2	0.29 ± 0.49^b^	2.71 ± 1.11	4.00 ± 0.81	1.53 ± 0.24^b^	0	0	7.50
E	PC21A	PC21A (P4); 12	26 (20)	0 (0/4)	0 (0/4)	7.24 ± 0.79^b^	–	0.00 ± 0.00	0.00 ± 0.00	0.00 ± 0.00	2.47 ± 0.47^a^	0	0	6.61
F^A^	Mock	PC21A (P2, P4); 10^A^, 12	29, 32 (25, 29)	100 (5/5)^A^	0 (0/5)^A^	10.57 ± 0.81^A^	1–2^A^	2.00 ± 0.71^a,A^	3.80 ± 0.84^A^	5.40 ± 0.55^A^	2.56 ± 1.51^A^	1–2^A^	4^A^	10.44

dpi, days post-inoculation; dpc, days post-challenge; RS, rectal swab score; NA, not available; GE, genomic equivalents.

^A^The first challenge on 29-day-old piglets did not induce clinical signs and viral shedding in naïve control pigs (litter F), so a higher dose was used. Evaluation of clinical signs of litters B and F was based on the second challenge at 32-days of age.

^B^RS score: 0, 1, 2, and 3 corresponded to normal, pasty, semi-liquid and liquid feces, respectively.

^C^ Virus passaged in gnotobiotic piglets (PC21A), frozen and thaw once, no filtration.

^a, b, c, d^Different letters in each column mean different levels among litters (*P* < 0.05). Pigs challenged with lower dose of the original PEDV (litter A) or at older ages (litter B and F) were excluded from statistical analysis.

Similarly, the dose of 10 log_10_ GE/pig of the original US PEDV PC21A caused no disease in pigs survived from S-INDEL PEDV Iowa106 infection (litter A). In litter B, only two piglets (Nos. 8 and 9) completely recovered from S-INDEL Iowa106 infection. Under the same schedule and doses as the mock-inoculated litter (litter F), pigs of litter B were challenged twice at 29 and 32 day-old. No clinical signs, but a slight increase of PEDV fecal RNA titer (7.44 ± 0.10 log_10_ GE/mL), was noted after the second challenge, but not after the first. In another two S-INDEL Iowa106-inoculated litters (litter C, *n* = 7; litter D, *n* = 7), pigs were challenged with 12 log_10_GE/pig of the original US PC21A. Those pigs developed diarrhea, lasting 2.29 ± 1.60 (litter C) and 2.71 ± 1.11 (litter D) days, respectively. Their body temperatures did not change after challenge. The median/mean fecal PEDV RNA shedding titers increased from 5.10 to 10.80 ± 0.76 log_10_ GE/mL in litter C and from <4.80 (detection limit) to 9.61 ± 1.64 in litter D at 1–2 dpc.

Pigs that survived the original US PEDV PC21A infection were challenged with the homologous strain (12 log_10_ GE/pig) at 25 day-old (21 dpi). No clinical signs were observed. The PEDV fecal RNA shedding titers increased slightly from 6.60 ± 0.50 (0 dpc/21 dpi) to 7.06 ± 0.82 log_10_ GE/mL during 3–5 dpc/24–26 dpi.

After piglets were challenged with the original US PC21A, only the PEDV naive sow F showed clinical signs, including anorexia for 2 days and diarrhea for 4 days. Her highest fecal PEDV RNA shedding titer was 10.44 log_10_ GE/mL. No clinical signs and lower (<8 log_10_ GE/mL) fecal PEDV RNA shedding titers were noted in the PEDV pre-exposed sows A–E.

All pigs were euthanized at 7 or 9 dpc. No significant gross and microscopic lesions were observed for all the litters. The VH:CD ratio ranged between 3.41 and 5.50 in jejunum and no significant differences were observed among the litters. By IHC staining, PEDV N proteins were detected in individual mononuclear cells in intestinal submucosa/Peyer’s patches and mesenteric lymph nodes in 75–100% of all pigs. On the other hand, only a few PEDV-positive epithelial cells at the villous tips (score = 1) were detected in the jejunum of pig No. 2 of litter E and in the ileum of pig No. 1 of litter F.

## Discussion

The first US S-INDEL PEDV strain, OH851, was identified in conventional pigs with minimal clinical signs and no mortality [15]. In the present study, the morbidity was consistently 100% in all S-INDEL PEDV Iowa106-inoculated piglets and 50% (2/4) in the contact exposed sows. High mortality (75%) was seen in one litter (litter B). The sows used in the present study were PEDV naïve until their piglets were inoculated. In the field observations, piglets in the farms may have been protected by PEDV-specific maternal antibodies since the sows were previously infected with S-INDEL PEDV OH851 strain [15]. Therefore, the different results between natural and experimental infections were likely due to the presence or lack of lactogenic immunity. Compared with a current study with one cell culture adapted, US S-INDEL PEDV strain USA/IL/2014/20697 [16], piglets in our study usually showed more pronounced clinical signs. Wild type or cell culture-adapted virus, doses of inoculum [10], environmental/animal conditions [19] and single nucleotide polymorphisms (SNPs) among US S-INDEL strains [8] could contribute to the variations observed among studies.

The S-INDEL PEDV strains that emerged in southern Germany showed high nucleotide identity (99.54%) in full-length genomes with US PEDV S-INDEL strains [17]. These German S-INDEL PEDV strains resulted in large variations in mortality (67.7% and 5.5% in 3-weeks-old and less than 1 week-old suckling piglets, respectively) in two farms [17]. Most recently, outbreaks of S-INDEL PEDV in Portugal were reported, causing severe diarrhea and high mortality [18]. Similarly, litter variations on the severity of PEDV infection were observed in our study, despite the similar background of sows and environmental factors. Among the S-INDEL PEDV Iowa106-inoculated piglets, the body weight of piglets measured on the day of inoculation (0 dpi) correlated negatively with the duration of diarrhea. In the original US PEDV PC21A-inoculated litter, five surviving piglets had significantly higher body weight at 0 dpi than their non-surviving littermates. In an large scale swine farm surveillance, lower piglet birth weight and higher within-litter variability of birth weight were the factors associated with higher losses from birth to weaning [26]. During PEDV infection, it is likely that the stronger piglets obtained more milk than their smaller littermates and were more likely to survive until intestinal villi regenerated and immunity developed. In a gnotobiotic mouse model, neonatal mice with better nutritional condition and higher body weight had higher enterocyte proliferation activity, more intensive response to probiotics and shorter duration of rotavirus-induced diarrhea [27]. In the present study, milk of sows provided the only food source for the piglets. Two of four sows of S-INDEL PEDV Iowa106-inoculation group showed diarrhea and anorexia, whereas the other two were asymptomatic. Since the sows’ health condition has a direct impact on the amount and quality of colostrum/milk [19], and it is critical to the infection outcome of their piglets. Based on our results, the severity of PED was associated with virus strain, piglet birth weight and sow health/lactation status. The impact of other factors, such as genetic background and gut microflora, requires further investigation.

The major target cells of PEDV are small intestinal epithelial cells. Previous histopathology studies demonstrated that a high percentage of villous epithelial cells in the small intestine was infected and destroyed by virulent PEDV strains shortly after clinical signs appeared [2, 5, 9, 28]. In both prototype PEDV CV777- and the original US PEDV US/Iowa/18984/2013A-inoculated CDCD piglets, PEDV antigen-positive enterocytes decreased from 1 to 2 dpi and then increased at 3–4 dpi [2, 11]. In the present study, we assessed the kinetics of virus growth in the intestine via quantitatively testing the daily rectal swab samples by RT-qPCR. In agreement with the above results, the first and also the highest peak of PEDV RNA fecal shedding titer was detected on the day of onset of clinical signs in both PEDV S-INDEL Iowa106- and the original US PC21A-inoculated litters. Afterward the titers of fecal PEDV RNA shedding decreased rapidly and then rebounded (Figure 1). Interestingly, the intervals (3–6 days) between fecal PEDV RNA shedding peaks were compatible with the reported typical replacement time of small intestinal villous epithelium in suckling piglets [29]. Since the replication of PEDV is sustained in enterocytes, this observation provided indirect evidence that both S-INDEL PEDV Iowa106 and the original US PEDV PC21A severely damaged the infected enterocytes and may spread to infect regenerating new enterocytes.

In the present study, inoculation of the original US PEDV PC21A to one piglet litter (*n* = 11) reproduced the results as described in our [9, 10, 12] and others’ [5, 13, 14] studies. Although piglet infection by S-INDEL PEDV Iowa106 also caused severe clinical signs in two litters (litter B and C), generally, the virulence of S-INDEL PEDV Iowa106 was lower than that of the original US PEDV strains as evident by: (1) a longer incubation time (delayed onset of clinical signs and the peak of viral RNA shedding); (2) a shorter duration of diarrhea; (3) relatively higher VH:CD ratios; (4) a lower percentage of PEDV-positive enterocytes; (5) more limited regions of virus infection (crypt not involved); and (6) overall lower piglet mortality (18 vs 55%) (Table 2). In addition, the profiles of fecal viral RNA shedding differed between the two PEDV strains. Typically, a biphasic curve with 3–6 day intervals between the two peaks (Figure 1C) was observed in S-INDEL Iowa106-inoculated piglets. On the other hand, a time-dependent and gradual downward-sloping curve (Figure 1D) was observed in the original US PEDV PC21A-inoculated piglets. These findings suggest that the replication kinetics of S-INDEL PEDV Iowa106 in piglets may be slower than that of the original US PEDV. The infection of S-INDEL is less severe than original US PEDV strains but varies, depending upon whether the sow also becomes ill with reduced milk production. Presumably, decreased pathogenicity of S-INDEL PEDV Iowa106 allowed time for the damaged intestinal villi to be re-populated with new enterocytes, leading to the survival of infected piglets.

In agreement with previous studies [2, 5, 10, 28], the original US PEDV infection was not restricted to epithelial cells covering the villi but, less frequently, also spread to some epithelial cells lining the crypts. In one piglet, infection by the original US PEDV PC21A was restricted to an individual crypt, but not observed in the adjacent villous epithelial cells (Figure 2C). Factors affecting the cell tropism of PEDV are still an important topic and need to be investigated in the future.

It is clearly established that the severity of PEDV infection is highly dependent on the age of pigs [12, 13, 25]. In the present study, one litter of mock-inoculated piglets was challenged with the original US PEDV PC21A (10 and 12 log_10_ GE/pig) at 29- and 32-days of age, respectively. However, only the repeated challenge with a 2 log_10_-higher dose induced mild clinical signs. In contrast, in the 3-day-old piglets (litter E), a lower dose (10 log_10_ GE/pig) of the original US PEDV PC21A could cause severe clinical signs. In one recent study, the minimal PEDV infectious dose in 5-day-old piglets was determined to be approximately 2 log_10_ lower than that in 21-day-old pigs [13]. The age-dependent resistance to PEDV infection [12, 13, 25] was again confirmed in this study. A longer time for replacement of villous epithelial cells [12, 29], a higher abundance of viral receptor expression [30] and the immature innate immune system in piglets less than 1-week of age have been proposed to explain the fatal TGEV or PEDV infection in young piglets. On the other hand, the sows were continuously exposed to high titers (>11 log_10_ GE/mL) of PEDV shed from their infected piglets. It is possible that lactating sows are more susceptible to enteric infection due to physiological changes associated with farrowing and lactation. In the present study, the sows had watery diarrhea. It is compatible to the previous field observations of sows during the original US PEDV [5] and Germany S-INDEL PEDV [17] outbreaks.

Current in vitro and in vivo studies suggest antigenic cross reactivity between the original US and S-INDEL PEDV strains [20, 21]. However, vaccines based on European and historic PEDV strains failed to control the more recent virulent PEDV outbreaks in Asia [19, 31], suggesting the possibility of antigenic variation among different PEDV strains [19, 20]. In the present study, a high dose (12 log_10_ GE/pig) of the original US PEDV challenge induced clinical signs in two S-INDEL PEDV Iowa106-inoculated pig litters (litter C and D), but not in the homologous strain-inoculated pig litter (litter E). An S-INDEL PEDV Iowa106-inoculated litter (litter A) challenged with 2 log_10_ lower dose of the original US PEDV PC21A (litter A) was excluded and no clinical signs were detected in these pigs. Our results suggest that the immunity induced by S-INDEL PEDV Iowa106 infection only partially protected pigs from the original US PEDV disease, which may result from antigenic variation between the original US and S-INDEL PEDV strains [19, 20]. However, other factors also need to be considered: (1) All piglets in litters C and D survived from S-INDEL PEDV Iowa106 infection and, subsequently, were challenged with the original US PEDV PC21A at 27-days of age. However, only 45% of piglets in litter E survived from the original US PEDV PC21A inoculation. These piglets had higher birth weights and may have been less affected by PEDV infection than their non-surviving littermates; (2) The effect of material antibodies may be affected by the titer of protective antibody in milk and the amount of milk, which sows provide to their piglets; (3) The level of acute immunity developed in the piglets can affect their susceptibility to repeated PEDV infection; and (4) The enterocyte turnover time is related to the age of pigs [29] and enteric viral infections [12]. Newly replaced villous enterocytes were speculated to be less susceptible to repeated PEDV or TGEV infection because innate and adaptive immune responses were elicited [32]. Studies of the detailed kinetics of PEDV humoral and cellular immune responses and the factors influencing the susceptibility of pigs to PEDV infection are ongoing and will be reported separately.

At the end of the study (28–30 dpi/7–9 dpc), the clinical signs and intestinal lesions subsided completely in all pigs. PEDV antigens were detected mainly in mucosal lymphoid tissues and mesenteric lymph nodes, but rarely in the villous epithelia of all recovered pigs. These PEDV IHC positive-stained mononuclear cells in lymph nodes were interpreted to be macrophages in recent studies [10, 11]. It is reported that piglets developed adaptive immunity around 7 dpi [14]. Our previous study showed that PEDV infection impaired the tight junctions of the villous epithelium [33]. PEDV-induced enteritis could attract macrophages to the gut. Development of mucosal immunity and increased permeability of the intestinal barrier could facilitate uptake of PEDV by macrophages and/or dendritic cells from the intestinal lumen.

In conclusion, our study suggests that S-INDEL PEDV Iowa106 is milder in virulence compared with the original US PEDV PC21A, but it still causes mortality in some litters. The severity of clinical signs induced by PEDV is associated with multiple factors, such as the birth weight of the piglets and the sow’s health/lactation status. In addition, a minority (19%, 3/16) of the piglets recovered from S-INDEL PEDV Iowa106 infection were fully protected from disease after a high challenge dose of the original US PEDV PC21A. Considering the safety and effectiveness, S-INDEL PEDV Iowa106 is not currently a suitable live vaccine to protect piglets from the highly virulent original US PEDV strains. Several original US PEDV strains, including PC21A, and S-INDEL PEDV Iowa106 strain have been successfully isolated from cell culture in our laboratory [23]. Evaluating the relatedness between cell culture adaption and in vivo virulence of these PEDV strains is ongoing for future attenuated vaccine development.

## Abbreviations

ANOVA: analysis of variance
ACOVA: analysis of covariance
CDCD: cesarean-derived, colostrum-deprived
dpc: day(s) post-challenge
dpi: day(s) post-inoculation
GE: genomic equivalent
Gn: gnotobiotic
IHC: immunohistochemistry
PBS: phosphate buffered saline
PED: porcine epidemic diarrhea
PEDV: porcine epidemic diarrhea virus
RS: rectal swab
RT-qPCR: quantitative real-time reverse transcription-PCR
S-INDEL: spike insertion and deletion
TGE: transmission gastroenteritis
TGEV: transmission gastroenteritis virus
VH:CD ratios: villous height and crypt depth ratios
